# A hepatitis A, B, C and HIV prevalence and risk factor study in ever injecting and non-injecting drug users in Luxembourg associated with HAV and HBV immunisations

**DOI:** 10.1186/1471-2458-11-351

**Published:** 2011-05-19

**Authors:** Nathalie Removille, Alain Origer, Sophie Couffignal, Michel Vaillant, Jean-Claude Schmit, Marie-Lise Lair

**Affiliations:** 1 Centre d'Etudes en Santé (CES), Centre de Recherche Public de la Santé, Luxembourg; 2 European Monitoring Centre for Drugs and Drug Addictions (EMCDDA) Focal Point Luxembourg, Centre de Recherche Public de la Santé, Luxembourg; 3 Service National des Maladies Infectieuses, Centre Hospitalier de Luxembourg, Luxembourg

## Abstract

**Background:**

In Luxembourg, viral hepatitis and HIV infection data in problem drug users (PDUs) are primarily based on self-reporting. Our study aimed to determine the prevalence of HAV, HBV, HCV and HIV infections in ever injecting (IDUs) and non-injecting drug users (nIDUs) including inherent risk factors analysis for IDUs. Secondary objectives were immunisation against HAV and HBV, referral to care and treatment facilities as well as reduction in risk behaviour.

**Methods:**

A nationwide, cross-sectional multi-site survey, involving 5 in-, 8 out-treatment and 2 prison centres, included both an assisted questionnaire (n = 368) and serological detection of HIV and Hepatitis A, B, C (n = 334). A response rate of 31% resulted in the participation of 310 IDUs and 58 nIDUs.

Risk factors such as drug use, sexual behaviour, imprisonment, protection and health knowledge (HAV, HBV status and immunisations, HCV, HIV), piercing/tattoo and use of social and medical services were studied by means of chi2 and logistic models.

**Results:**

Seroprevalence results for IDUs were 81.3% (218/268, 95%CI=[76.6; 86.0]) for HCV, 29.1% (74/254, 95%CI=[25.5;34.7 ]) for HBV (acute/chronic infection or past cured infection), 2.5% (5/202, 95%CI=[0.3; 4.6]) for HIV-1 and 57.1% (108/189, 95%CI=[50.0; 64.1]) for HAV (cured infections or past vaccinations). Seroprevalence results for nIDUs were 19.1% (9/47, 95%CI=[7.9;30.3]) for HCV, 8.9% (4/45, 95%CI=[0.6;17.2]) for HBV (acute/chronic infection or past cured infection), 4.8% (2/42, 95%CI=[-1.7;11.3]) for HIV-1 and 65.9% (27/41, 95%CI=[51.4;80.4]) for HAV. Prisoners showed the highest rates for all infections. Age, imprisonment and setting of recruitment were statistically associated with HCV seropositivity. Age, speedball career and nationality were significantly associated with HBV seropositivity. Only 56% of the participants in outpatient centres collected their serology results and 43 doses of vaccine against HAV and/or HBV were administered.

**Conclusions:**

Despite the existing national risk-reduction strategies implemented since 1993, high prevalence of HCV and HBV infections in injecting drug users is observed. Our study showed that implementing risk-prevention strategies, including immunisation remains difficult with PDUs. Improvement should be looked for by the provision of field healthcare structures providing tests with immediate results, advice, immunisation or treatment if appropriate.

## Background

The prevalence of hepatitis C virus (HCV) infection in injecting drug users varies between regions in Europe from 40 to 90% [1]. Before 2005, data on the prevalence of hepatitis B virus (HBV) infection in IDUs were limited. Prevalence figures for Europe in 2007-08 [2] were highly variable due to differences in vaccination coverage. The positivity of the Hepatitis core antibody (anti-HBc) which indicates a contact with the viral particle reveals a hepatitis B prevalence over 40%. Limited prevalence data exist for hepatitis A (HAV) infection in the same population. Human immunodeficiency virus type 1 (HIV-1) prevalence in IDUs ranges between 0% and 21% in the European Union, although it does not exceed 5% in most Member states [3].

Prevalence of HCV among nIDUs varies in Europe between 10 and 20% [4] and remains higher than among the general population. The same prevalence trend is observed among nIDUs for HBV and HIV infections [4-6].

Epidemiological follow-up of problem drug use in Luxembourg relies on a nation-wide multi-sector surveillance system called "RELIS". RELIS was established in 1995 and conceived on the methodological assumption that data exclusively collected from drug treatment settings may not provide an accurate picture of the problem drug-using population as it notably excludes out-of-treatment drug users who for instance have had conflicts with law enforcement bodies due to the problematic aspect of their drug use. The RELIS network includes in- and out-patient treatment centres (100% coverage), including hospitals providing detoxification treatment, national prisons and law enforcement agencies. Data collection is performed via a series of contextual protocols ("first contact", "update" or "identification" protocol), self-reported data and setting-related data (number of: contacts, syringes exchanged...). The yearly output of RELIS largely contributes to the publication of the national annual drugs report. According to RELIS data [3] a weak upward trend in HIV/IDUs self-reported prevalence has been observed from 3.5% in 1998 to 5% in 2004, and hepatitis C prevalence stood at 74% in 2004. In 1998, the first published data about Luxembourg prison [7] reported HIV and HCV prevalence in IDU prison inmates of 4.3%, respectively 37%. Among all reported new cases of HIV-1 infection in Luxembourg, few seem to occur in IDUs [4]: 6 HIV-1 infections out of 30 new cases (20%) occurred through injecting drug use in 1999 compared to only 3 out of 60 (5%) in 2004 [8].

Our study pursued three goals: i) defining the prevalence of viral hepatitis B, C and HIV in IDUs and nIDUs in Luxembourg, ii) analysing risk factors of viral infections in IDUs and iii) assessing the need and feasibility of HAV and HBV immunisations in PDUs.

## Methods

### Settings and participants

To approach the genuine heterogeneity of the drug misuse phenomenon, RELIS routinely compiles data from law enforcement agencies, the 2 prisons, inpatient (specialised or psychiatric hospital wards: 6) or outpatient (substitution treatment programmes, low-threshold facilities, drop-in centres for sex workers, drug consultation centres: 8) centres providing counselling, assistance and drug treatment to PDUs throughout the country. Also, the national drug surveillance system relies on the concept of the 'institutional contact indicator', as an alternative to the better-known treatment demand indicator [9]. Furthermore, the case definition applied by RELIS, detailed below, refers to PDUs and is not limited to IDUs, heroin users or opiate users - even though the RELIS database allows extracting data on these sub-groups.

According to the RELIS case definition, a problem user of illicitly acquired drugs (referred to as PDU) is defined as a person who shows a) current and regular use of opiates, cocaine, and/or amphetamines, and b) current contact with a health or law enforcement institution due to the use of listed drugs. Route of administration is not considered as a selection criteria. The use of other licit or illicit substances whether prescribed or not, is not an exclusion criteria as long as it is associated with the described drug use pattern.

The latest PDU prevalence study prior to the present research reported an absolute prevalence of 2530 PDUs among the national population aged 15 to 64 years [7]. To reach representativeness of the PDUs population in Luxembourg, a sample of 400 respondents (15-16%) was projected, aiming for similar rates of recruitment in all participating sites. The study sites included inpatient (ITC) and outpatient (OTC) treatment centres and prisons (PC) in Luxembourg. ITCs are centres where PDUs have spent at least one night (e.g hospitals). OTCs are settings where ambulant PDUs and sex workers can have easy and free access to drug substitution, psychological or medical advice, as well as services such as exchange programmes or social counselling. Basic physical and psychological needs are also addressed in such settings.

Among the 16 eligible sites (2 PC, 8 OTC, and 6 ITC) and leaving aside law enforcement agencies and one outpatient centre for minors only, the study sites included all the RELIS network centres with the exception of one hospital. This allowed reaching remarkable national service representativeness.

PDUs in contact with participating institutions were eligible for inclusion in the study when reporting use of opiates, cocaine, amphetamines or being under medical substitution (methadone, buprenorphine...) irrespective of the route of administration and duration of use. IDUs are defined as having injected at least once in lifetime.

The study was designed as a "research-action", offering participants free medical counselling, risk-reduction material and immunisation against HAV and HBV. The study was performed according to the Declaration of Helsinki and approved by the National Research Ethics Committee and the National Commission for Data Protection (NCDP).

### Questionnaire

The trained field investigator informed each participant on the study before proceeding to an assisted questionnaire and a prevention message with handover of risk reduction material. A personal identification code (RELIS code) approved by the NCDP, was computed from gender, date and country of birth variables.

Written informed consent was obtained for all interviews, blood sampling or medical file consultation. Each stage of participation gave right to a free meal voucher for the participants. Multiple strategies were used to facilitate recruitment such as flyer distribution (central railway station, pharmacies...) and direct approaching of PDUs by street workers.

The questionnaire was based on a "Scottish questionnaire" [10] and the EU consensus questionnaire designed by the European Monitoring Centre for Drug and Drug Addictions (Van Ameijden E, Wiessing L: *Consensus questionnaire for Young Problematic Drug Users. Lisbon EMCDDA meeting 2000 jun 15-16*. Unpublished reports.) focusing on socio-demographic information, drug use, injecting behaviour, sexual behaviour, imprisonment, protection and health knowledge (HAV, HBV status and immunisations, HCV, HIV), piercing/tattoo and use of social and medical services. This questionnaire was made available in French, German and Portuguese after a validated translation procedure. On average, it took 30 minutes to be completed.

### Blood sampling

In ITCs and PCs, serology results and immunisation follow-ups were gathered from medical files. In OTCs, PDUs were offered blood sampling for HIV and hepatitis serology. During the 8-month recruitment period and for an additional 6 months after the inclusion of the last respondent, results and, if needed, immunisations were given at the same location by a physician. Blood samples collected on site were transported to the laboratory on the same day. Serum specimens were aliquoted and stored at -20°C until the ELISA serology tests were performed on an automated Abbott AXSYM System (Abbott, Brussels, Belgium). The following tests were performed: for HAV, IgG (HAVAB 2.0 Reagents) and IgM antibodies (HAVAB.M 2.0 Reagents), for HBV, HBs antigen, anti-HBs antibodies (AUSAB reagents), anti-HBc antibodies (CORE reagents), for HCV, anti-HCV antibodies (HCV 3.0 reagents), and for HIV-1 and 2 (HIV Ag/Ab Combo Reagents). In case of a positive ELISA test for HCV or HIV, confirmation tests were manually performed (for HCV, CHIRON RIBA HCV 3.0 SIA (Ortho Clinical Diagnostics, Raviatan, New Jersey, USA) and for HIV, HIV BLOT 2.2 (Genelabs Diagnostics, Singapore, Singapore) and Vironostika HIV-1 Antigen (Biomerieux, Boxtell, The Netherlands)).

### Immunisations

When appropriate, immunisations against HAV and HBV with Havrix 1440, Engerix B20, or Twinrix Adult (GlaxoSmithKline, Rixensart, Belgium) were offered free of charge in OTC. Immunisations were provided routinely - independently of the present study - in ITC and PC.

### Statistical analysis

The use of the RELIS code coupled with a single field investigator allowed avoiding duplicates in the process of data management. Student T-tests compared continuous variables, Chi-square or Fisher's exact tests where appropriate compared categorical variables. Odds ratio and their Confidence Intervals (95%CI) were calculated when possible. For both HCV and HBV, logistic regression was applied by using a stepwise manual backward approach to select probable risk factors (that were significant at a p-value below 0.20 in descriptive analysis) where a non-significant likelihood ratio test was found. The final model was also adjusted for known covariates (age).

A p-value below 0.05 was considered statistically significant. All tests were two-tailed. Analyses were carried out with SPSS Statistical software version 13 (SPSS Inc. Chicago, IL, USA) and SAS System version 9.2 (SAS Institute, Cary, NC, USA).

## Results

### Description of the study population

**Figure 1:**
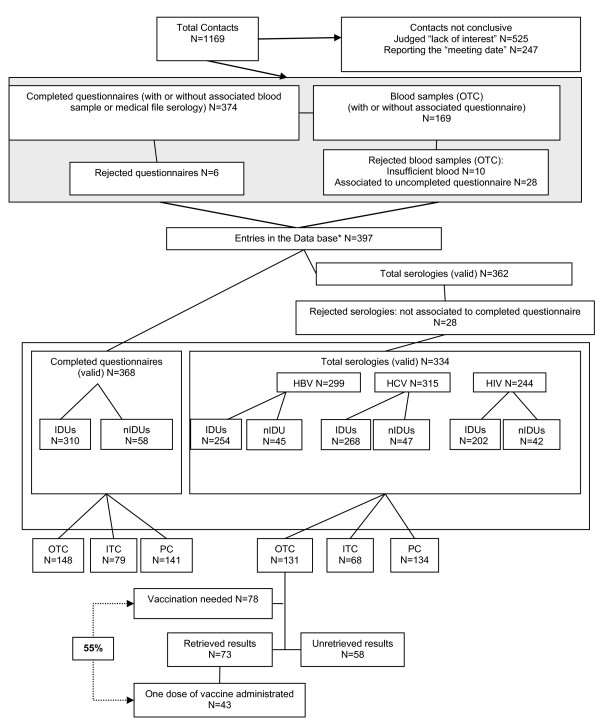
**Study Flow Chart**. * Respondents could either do both the questionnaire and the screening/give access to their serology or just one of these two.

From January 1 to August 31, 2005, 1,169 contacts were made with PDUs (Figure 1). The main causes of participation refusal was "lack of interest" in the study (N = 525) and some participants refused to participate simply by postponing questionnaire completion without showing up again (N = 247). Tests on internal consistency rejected 6 questionnaires for contradictory answers, 10 blood-screening samples yielded insufficient amounts of blood and 28 serologies were excluded because not coupled with a completed questionnaire. Serology results from ITC and PC were included in the analysis only if obtained between January 1, 2005 and August 31, 2005, unless they had already been positive for HAV, HBV, HCV or HIV before January 2005. Finally, 368 inclusions were accepted (participation rate: 31%), representing about 14.5% of the estimated RELIS 2005 [11] total PDU population in Luxembourg, of whom all completed the questionnaire and 334 had valid serology results.

Participation rate of PDUs in the questionnaire-based interviews was lower in OTCs (148/176; 84%) than in ITCs (79/79; 100%) and PCs (141/142; 99%) but no difference in the distribution of IDUs and nIDUs (non-IDUs) between the 3 settings (OTC 119/148, ITC 69/79, PC 122/141, p = 0.25) was observed. 84% (310/368, 95%CI=[80.2; 87.7]) of respondents were IDUs and 16% nIDUs, constituting the studied population whose main characteristics are displayed in Table 1. Men were significantly (p = 0.035) older (mean +/- SD: 31 +/- 7.9 years) than women (29 +/- 6.4) in IDUs and nIDUS (men 29 +/- 7.5 vs women 24 +/- 8.7, p = 0.029). Women in IDUs were older (29 +/- 6.4) than among nIDUs (24 +/-8.7) (p = 0.021).

**Table 1:** Main characteristics of the study population at inclusion (N = 368)

Parameter (N = 368)	Category	IDUs N (%)	nIDUs N (%)
Gender	Male	248/310 (80.0)	47/58 (81.1)

Nationality	Luxembourg	183/310 (59.0)	24/58 (41.4)

	Portugal	60/310 (19.3)	13/58 (22.4)

Children at charge	Yes	54/310 (17.4)	9/58 (15.5)

Life time drug use	Heroin	279/279 (100.0)	28/28 (100.0)

	Cocaine	280/310 (90.3)	37/58 (63.8)

	Cannabis	286/334 (85.6)	48/334 (14.4)

	Speedball (heroin/cocaine combination)	205/310 (84.2)	0/58 (0.0)

Last 6 months IDU	Never borrowed used paraphernalia	162/256 (63.3)	

	Never lend used paraphernalia	161/255 (62.9)	

	Mostly shared: cups	69/94 (73.4)	

	Mostly shared: filters	68/94 (72.3)	

	Mostly shared: syringes	42/94 (44.6)	

Last 6 months number of sexual partners (N = 266)	None	192/308 (62.3)	32/58 (55.2)

	1-9	94/308 (30.5)	26/58 (44.8)

	10+	22/308 (7.1)	0/58 (0)

Imprisonment (N = 366)	At least once during the last 10 years	215/308 (69.8)	31/58 (53.4)

Drug consumption in prison (N = 246)	Yes	130/215 (60.5)	8/31 (25.8)

Substitution treatment (N = 368)	Yes	286/310 (92.3)	19/58 (32.8))

Tattoo or piercing	None	87/309 (28.2)	28/58 (48.3)

	Tattoo	100/309(32.4)	14/58 (24.1)

	Piercing	23/309 (7.4)	9/58 (15.5)

	Both	99/309 (32.0)	7/58 (12.1)

Note: Denominators may vary because of missing answers to some questions.

Among IDUs the average number of years elapsed since the first use of injectable drug (cocaine, heroin, speedball, amphetamines) was 10.0 years (SD: 7.5). From the IDUs having injected in the last 6 months, 75% of the sterile syringes were obtained through syringe exchange programs and 12% from pharmacies. Sixty-five percent gave back their used syringes at a syringe exchange program point, although 15% of these syringes were separated from their needle and 8% of the needles were discarded in an inappropriate location (e.g. dustbin or street).

In prisons, 75 in 122 IDUs (55%) injected drugs during their detainment. Forty of them (53%) reused their own syringes and needles which had never been shared with another person. Among the 60 IDUs who reused their syringes whether shared or not, 43 (71%) cleaned them with water only.

### Prevalence of infections

HBV serology was considered positive (HBVab) - either acute or chronic active or past cured infection in 29.1% (95%CI: 25.5-34.7) of IDUs and 8.9% (95%CI: 0.5-17.2) in nIDUs -including 18 anti-HBc antibodies-only cases (Table 2). Among IDUs, 3.9% (95%CI: 1.5-6.2) showed acute or chronic HBV infection (i.e. HBs antigen positive) where nIDUs showed none. Seroprevalence for HCVab was 81.3% (95%CI: 76.6-86.0) and 19.1 (95%CI: 7.9-30.3) for nIDUs. HIVab seroroprevalence was 2.5% (95%CI: 0.3-4.6) in IDUs and 4.8% (95%CI: -1.7-11.3) in nIDUs. Among IDUs HAVab was 57.1% (95%CI: 50.0-64.1) and 65.9% (95%CI: 51.4-80.4) among nIDUs. No acute cases of HAV infection (i.e. IgM positive) were detected. Seroprevalence according to study subpopulations are outlined in Table 2.

**Table 2:** Seroprevalence (%)

		OTC N/Ntotal	ITC N/Ntotal	PC N/Ntotal	IDUs N/Ntotal	nIDUs % N/Ntotal
	Active HBV (HBs ag+)	1.5 2/130	0 0/54	7.0 8/115	3.9 10/254	0 0/45
	
HBV (cured or active infection)	Cured HBV (HBs ab+, HBc ab+)	13.1 17/130	14.8 8/54	23.5 27/115	19.3 49/254	6.7 3/45
	
	Total HBVab *	22.3 29/130	16.7 9/54	34.8 40/115	29.1 74/254	8.9 4/45

HBV vaccination (HBs ab+)		39.2 51/130	57.4 31/54	45.2 52/115	46.1 117/254	37.8 17/45

HBV seropositivity (all types)		61.5 80/130	74.1 40/54	80.0 92/115	75.2 191/254	46.7 21/45

HCV (Elisa +, RIBA +) HCVab		57.3 75/131	75.4 46/61	86.3 107/124	81.3 218/268	19.1 9/47

HAV (IgG+) HAVab		54.7 70/128	57.1 24/42	68.3 41/60	57.1 108/189	65.9 27/41

HIV-1 HIVab		1.5 2/130	0 0/49	7.7 5/65	2.5 5/202	4.8 2/42

*Including 18 cases with HBc antibody only.

Compared to nIDUs, IDUs show higher rates for HBVab (p = 0.0038) and HBV non- vaccinated status (p = 0.0001). There is no statistical difference between IDUs and nIDUs for HBV vaccinated status (p = 0.30), HAVab (p = 0.31) nor HIVab (p = 0.43), but HCVab is higher in IDUs (OR: 18.4, 95%CI=[8.4; 40.5]).

Among participants with HCVab, HBVab or HIVab, 96% (218/227), respectively 95% (74/78), and 71% (5/7) were IDUs.

### Co-infections

Among HCV positives, 61.0% (94/154, 95%CI=[53.3; 68.7]) were protected against HBV either by vaccination (40.3%, 62/154, 95%CI=[32.8; 48.2]) or past infection (20.8%, 32/154, 95%CI=[15.1; 27.9]). All 7 respondents with active HBV infection and all 7 HIV-1 positives were co-infected with HCV.

### Covariates of infection: univariate and multivariate analyses (Table 3 and 4)

In IDUs, HCV (Table 3) and HBV (Table 4) infections were significantly associated with increasing age and duration of drug use for heroin, cocaine and speedball.

**Table 3:** Covariates of HCV infection in IDUs (univariate analysis)

Parameter	Category	N per category	HCV + N (%)	OR	95%CI
Age, years (N = 268)	18-24	61	36 (59.0)	1.0	

	25+	207	182 (87.9)	5.0	[2.6; 9.8]

Settings (N = 268)	OTC	103	74 (71.8)	1	

	ITC + PC	165	144 (87.3)	2.7	[1.4; 5.0]

Heroin career, years (N = 239)	0-5	51	33 (64.7)	1	

	6+	188	166 (88.3)	4.1	[2.0; 8.5]

Cocaine career, years (N = 240)	0-5	89	64 (71.9)	1.0	

	6+	151	131 (86.8)	2.6	[1.3; 4.9]

Cannabis, years (N = 248)	0-5	36	24 (66.7)	1.0	

	6+	212	182 (85.8)	3.0	[1.4; 6.7]

Speedball career, years (N = 179)	0-5	111	84 (75.7)	1.0	

	6+	68	63 (92.6)	4.1	[1.5; 11.1]

Last heroin consumption, m:month (N = 267)	0-1m	155	117 (75.5)	1	

	2m +	112	100 (89.3)	2.7	[1.3; 5.5]

Last amphetamine consumption y:year (N = 164)	1y	29	18 (62.1)	1	

	2 y +	135	111 (82.2)	2.8	[1.2; 6.7]

Sex partners, number in last 6 months (N = 267)	None	162	139 (85.6)	1	

	1+	105	78 (74.3)	0.5	[0.2; 0.9]

Substitution treatment, (N = 268)	No	22	13 (59.1)	1.0	

	Yes	246	205 (83.3)	3.5	[1.4; 8.6]

Imprisoned, times (N = 267)	0-1	197	153 (77.7)	1	

	2+	70	64 (91.4)	3.1	[1.2; 7.6]

Drug use in prison, (N = 189)	No	75	55 (73.3)	1	

	Yes	114	103 (90.3)	3.4	[1.5; 7.5]

**Table 4:** Covariates of HBV infection in IDUs (univariate analysis)

Parameter	Category	N per category	HBV + N (%)	OR	95%CI
Age, years (N = 254)	18-24	8	12.9	1	

	25+	66	34.4	3.5	[1.6; 7.8]

Nationality (N = 254)	Other	21	39.6	1	

	Portugal	20	38.5	2.2	[1.1; 4.3]

	Luxembourg	33	22.1	2.3	[1.2; 4.5]

Heroin career, years (N = 227)	0-9	21	22.8	1	

	10+	51	37.8	2.1	[1.1; 3.7]

Cocaine career, years (N = 229)	0-5	17	19.8	1	

	6+	51	35.7	2.2	[1.2; 4.2]

Speedball career, years (N = 168)	0-5	22	21.4	1	

	6+	30	46.2	3.2	[1.6; 6.2]

Amphetamine carreer, years (N = 158)	0-5	22	20.4	1	

	6+	19	38.0	2.4	[1.1; 5.0]

HCV infection was associated with type of setting (ITC and PC), substitution treatment history and multiple stays in prison or drug use in prison. None of the disinfection procedures in prison (water, chlorine, alcohol, burned needle, aftershave...) correlated significantly with HCV. The duration of drug use for cannabis and a distant date (see Table 4 for duration from last drug use) of last heroin or amphetamine use was statistically associated with HCV infection.

HBV infection was significantly associated with nationality (Portugal) and amphetamine career in IDUs.

In order to assess the risk factors of the different infections, we entered in the multivariate models variables with a p-value lower than 0.20 in the univariate analysis.

For HBV infection in IDUs, multivariate logistic modeling discarded cocaine, heroin and amphetamine careers. The final model included age (≥25y versus ≤24y: OR = 2.7, 95%CI=[1.2;6.2]), speedball career (≥6y versus ≤5y: OR = 2.3, 95%CI=[1.1;4.4]) and nationality (other vs Luxembourg: OR = 2.0, 95%CI=[1.0;4.1]) as the only significant factors. Age did not appear as a factor of interaction for speedball career (p = 0.66) and the model was adjusted to avoid any confusion between HBV infection and speedball career.

Regarding HCV, the multivariate modeling discarded cocaine, heroin, speedball and cannabis careers, last heroin and amphetamine consumption, substitution treatment, sex partners, imprisonment and drug use in prison. The final logistic model for HCV infection in IDUs included age (≥25y vs ≤24y: OR = 4.2, 95%CI=[2.1;8.6]) and the settings of recruitment (ITR+PC vs OTC: OR = 2.3, 95%CI=[1.2; 4.6]) as the only significant factors. Age appeared as a confounding factor for drug use careers (p = 0.0001).

### Self-reporting of infections and immunisations

Comparability and validity of self-reported study results and respective RELIS data, including sensitivity, sensibility and agreement analysis have been specifically addressed in a former research paper [12].

As far as the infection status awareness of PDUs is concerned, respectively 81 and 59 PDUs out of 360 did not know about their past 10 years acquired HAV or HBV immunisation. Respectively 131 and 120 PDUs self-declared not having been vaccinated against HAV or HBV.

Of 148 and 181 respondents recalling previous HAV and HBV vaccination respectively, 22% (33/148) and 20% (36/181) reported one single dose of vaccine, 20% (29/148) and 16% (29/181) two doses, 47% (69/148) and 55% (99/181) three doses of vaccine; 17 PDUs (11% HAV, 9% HBV) did not know the number of administrated boosters.

### Hepatitis A and/or B immunisations

Protection against HBV was either acquired by past infection (19.3%, 95%CI=[14.4; 24.2] for IDUs, 6.7% 95%CI=[-0.6; 14.0] for nIDUs), or by vaccination (46.1%, 95%CI=[40.0; 52.2] for IDUs, 37.8% 95%CI=[23.6; 52.0] for nIDUs) (Table 2). 26.4% (63/239; 95%CI=[20.8-32.0]) of IDUs, 54.5% (24/44; 95%CI=[39.8-69.2]) of nIDUs were still susceptible to HBV infection and hence eligible for HBV immunisation. 43% (81/189; 95%CI=[35.8-50.0]) of IDUs, 34% (14/41; 95%CI=[20.0-48.6]) of nIDUs were not immunised against HAV (Table 2). There was no statistical difference in HBV vaccination rates between the 3 settings (p = 0.154). However, from 131 PDUs tested in OTC, only 73 (55%) returned to collect their results (Figure 1). Of the latter, 39% (17/43) received one Twinrix^® ^dose, 32% (14/43) one Havrix^® ^dose and 28% (12/43) one Engerix^® ^dose. Of the 123 interpretable blood samples, respectively 28 (23%), 30 (24%), 20 (16%) PDUs were potential candidates for vaccination against hepatitis A, hepatitis A and B and hepatitis B. Fifty-seven percent of PDUs requiring HAV and HBV immunisation received one Twinrix^® ^dose, 50% requiring HAV immunisation received one Havrix^® ^dose and 60% requiring HBV immunisation received one Engerix^® ^dose. In OTC, 78 (52%) respondents needed immunisation (HBV and/or HAV) and 43 (55%) received a first dose of vaccine. No second dose of vaccine was administered, as none of the PDUs showed up for a booster.

## Discussion

Our work aims to provide a comprehensive nationwide insight into selected chronic viral infections in IDUs and non IDUs. All types of PDU care settings were included in the study and about 15% of the estimated PDU population in Luxembourg was recruited with a low participation rate of 31% following initial contact. Part of the explanation may lie within the recruitment in low-threshold services with PDUs often deferring participation and not reappearing later.

A comparison between respondents and non-respondents in this hard-to-reach population was not possible in our study. However, we gathered the most representative sample of the Luxembourg PDU population although a selection bias stands with lower participation rate in outpatients (84%) and higher ones in inpatients (100%) and prisons (99%). A cross-sectional study conducted in Luxembourg in 1999 also showed a 33.9% response rate [13]. Other limitations of the study can be detected in the recruitment method targeting exclusively the PDUs in contact with ITCs, OTCs or PCs, thereby not reaching those not in contact with such institutions. Secondly, the heterogeneity of the types of PDUs (injectors or not) attending the various settings makes the interpretation of the results somewhat uneasy. Since the initiation of the project had an action-research design, the first priority was to reach, test and treat if required as many PDUs as possible, which implies less selective recruitment criteria. Also, the questionnaire items did not allow differentiating between ever- and current injectors, which would have allowed further in-depth analysis.

Overall, HCV prevalence was 81.3% in IDUs and 19.1% in non IDUs. Those rates put Luxembourg in the upper range of HCV prevalence among IDUs in Europe [1]. Among nIDUs the rate is lower compared to Italy or Spain [5,6], but remains much higher than the estimated HCV prevalence rate (0.5%-1%) in the general population [14].

Compared to PCs (86.3%; 95%CI: 80.2-92.3), the prevalence of HCV was lower in ITCs (75.4%; 95%CI: 64.6-86.2) and OTCs (57.3%; 95%CI: 48.8-65.8). There may be a selection bias in recruitment as outpatients showed less motivation to participate in the study by postponing their participation. Also, in prison the access to sterile material brings IDUs to be more at stake when it comes to infection risks.

Alike, Belgium [15] reported higher HCV prevalence rates in imprisoned drug injectors (76%). In the HCV multivariate analysis, being an IDU older than 25 years and being in ITC or PC rather than in OTC increased the risk (OR: 4.2) of HCVab. Consistent with other studies, each year of injection drug use increases the risk of contracting HCV by a factor of 1.14, and drug use in prison generates a risk of 3.16 fold [10,16].

Since no viral load assays were performed in our study, we were unable to distinguish chronic active from past resolved HCV infections. Based on literature, we can estimate however that about 55-85% of seropositive had indeed an ongoing chronic infection [17]. In addition, a viral genotype assay might have added valuable information. A recent study performed in Luxembourg suggests that IDUs are 4.54 times more likely to be infected by HCV genotype 3 than other patients, even if adjusted for age and gender [14]. Genotype 3 has roughly 80% of chances of sustained response to a standard 6-month interferon/ribavirin treatment. Preliminary data now show that a shorter treatment course of only 3 months may have the same cure rate [18]. Therefore, one might speculate that offering short antiviral treatments to a large number of IDUs with genotype 3 would have the potential to reduce the transmission rate in the population by dramatically lowering the infectious reservoir.

In IDUs, HBVab prevalence was 29.1%. Again we found significantly higher HBVab prevalence in prisons (34.8%;95%CI: 26.1-43.5) compared to other settings. In IDUs, national prevalence rates for HBcAb, HBsAg and HBsAb are scarce in Europe, ranging respectively from 0.7%-64%, 2.3%-6.1% and 25%-37% [19]. Belgium reports 18% HBVab (acute/chronic infections and cured infections) in IDUs [15]. Our study shows high HBsAb (65.4%) probably due to a relatively high HBV vaccination rate in IDUs when compared to other countries [20,21]. Also, only about 3.9% (95%CI: 1.5-6.3) of IDUs presented active HBV infection (Antigen HBs positive), leaving little room for therapeutic interventions. On the other hand, with only 46% of IDUs (95%CI: 40.0-52.2) protected by vaccination and 19.3% (95%CI: 14.5-24.2) having acquired HBV, there is clearly a need for an improved vaccination policy at an earlier stage. Among nIDUs, HBVab prevalence was 8.9%, showing a lower rate than Italy or Spain (respectively 22.8%, 20.7%) [5,6]. In the HBV multivariate analysis, increasing age, more than 6 years' speedball career and non-Luxembourg nationality increases the risk of contracting HBV.

The results of the multivariate analysis with increasing age, more than 6 years' speedball career and non-Luxembourg nationality favouring HBV transmission are consistent with those of other studies [20,22]. They concur with the need to improve an early stage vaccination policy and the one to target non-nationals.

Vaccinations for PDUs are recommended in all the settings wherever feasible and although no difference in HBV vaccination rates according to different settings has been observed, awareness should be further raised among physicians regarding the importance and usefulness of HBV vaccination in PDUs.

We found no case of acute HAV infection; however, 41% of PDUs were unprotected against HAV and would have benefited from immunisation.

The prevalence of HIV among IDUs was 2.5%. Again the highest prevalence was found in prison (7.7%). In the European Union, HIV prevalence in IDUs varies from 1% in the UK to 30% in Spain [23]; Neighbouring Belgium reports 3% and 4% in prisons among IDUs [15]. The lower rates for HIV infection compared to HCV might be explained by lower viral infectivity [24,25]. Among nIDUs, HIV prevalence was 4.8% showing a higher rate than in Italy (1.6%) and Spain (2.7%) [5,6]. This rate is also higher than the estimated 0.2% HIV infection rate in the general population of Luxembourg. Nevertheless, the highest risk of HIV contamination in Luxembourg, remains through male homosexual intercourse or heterosexual contacts [26].

Our data shows that co-infections are common. The univariate analysis in the IDU population associates gender with HBV, age with HBV and HCV, and nationality with HCV infection. Likewise findings from other studies [20,21] showed that duration of drug use was significantly associated with an increase in HBV and HCV infections (Table 3).

Drug injectors are particularly exposed to infection risks [27,28] as they are apt to lend or borrow paraphernalia. Cups and filters were the most commonly exchanged objects in our study, even though free of charge, sterile and ready-to-use kits for drug use are widely available in the vicinity of the main consumption spots. Sixty-five percent of drug-injecting study respondents reported discarding their used syringes in syringe exchange programmes. Worrisome, however, is that syringes are inappropriately eliminated in 23% of the cases with a syringe/needle separation occurring in 15% and needles discarded in an inadequate place in 8% of the cases. These types of behaviour result in increased infection risks for others. Cleaning or disinfection of used syringes and needles is predominantly observed in prisons. Yet, 72% use only water (which does not disinfect) for cleaning, which points out to the fact that appropriate prevention measures were not readily available in prisons. As lifestyle and consumption habits of PDUs as well as daily constraints to procure drugs rarely comply with appointments, the organisation and maintenance of medical interventions were highly challenging. As long as the treatment centres' employees and the field investigator who had established close contacts with the participants, reminded them of their appointments, about half (54.6%) of the tested PDUs saw the physicians and received immunisations where appropriate. After the 8-month inclusion period, only the physicians were active in the field in order to complete the immunisation programmes. Although the system was meticulously planned, not one additional follow-up was obtained under these conditions. One way of improvement might be rapid, point-of-care tests providing results in a few minutes and allowing immediate advice and immunisation if appropriate. However, even if completing a vaccination scheme was the ultimate goal, one has to bear in mind the concept « One dose is better than no dose » [29], which suggests that there is a protective effect even from uncompleted schemes.

## Conclusions

In conclusion, our findings confirm that the prevalence of chronic viral infections and especially HCV is high among IDUs in Luxembourg. Also, the prevalence among nIDUs is noticeable for HBV. Therefore low- threshold opportunities for immediate testing, information on prevention, access to prevention material, immunisation and treatment if appropriate should be improved among PDUs. A syringe distribution programme for IDUs in prisons is highly suggested and has been in force in Luxembourg since the summer of 2005. Awareness should be further raised among physicians regarding the importance and usefulness of HBV/HAV vaccination in PDUs at the earliest stage of PDU. Tackling the problem of chronic viral infections in PDU is difficult, especially when it comes to gaining adherence to screening and immunisation schemes as shown in our study. In order to evaluate future trends in viral infections and effects of prevention programmes, the implementation of a continuous surveillance system for chronic viral infections in the PDU population should be considered [30,31].

## Abbreviations

PDUs: Problematic Drug Users; IDUs: Injecting Drug Users; nIDUs: non Injecting Drug Users; HAV: Hepatitis A Virus; HBV: Hepatitis B Virus; HCV: Hepatitis C Virus, HIV: Human Immunodeficiency Virus; OR: Odds Ratio; OTC: Out Treatment Centre; ITC: In Treatment Centre; PC: Prison Centre; IgG: Immunoglobulin G, IgM: Immunoglobulin M, NCDP: National Commission for Data Protection.

## Competing interests

The authors declare that they have no competing interests.

## Authors' contributions

All authors read and approved the final manuscript. AO developed the concept and tendered for funding. NR and AO developed the protocol and reported the study. NR supervised the fieldwork, the data collection and the data entry. NR, AO, SC contributed to the analysis plan. NR carried out the analysis. MV supervised the statistical analysis. MLL and AO supervised the implementation of the study. JCS supervised the development of laboratory methods and laboratory analysis and was a reference physician on the fieldwork. JCS, MV, AO, MLL and SC reviewed different versions of the manuscript. NR wrote the first draft and amended subsequent versions.

## Pre-publication history

The pre-publication history for this paper can be accessed here:

http://www.biomedcentral.com/1471-2458/11/351/prepub
